# Supplementary material for: Extreme fast charging of commercial Li-ion batteries via combined thermal switching and self-heating approaches
Source: Nat Commun. 2023 Jun 3;14:3229. doi: 10.1038/s41467-023-38823-9 (PMC10239438; doi:10.1038/s41467-023-38823-9)
Supplement: Supplementary file 1 — Supplementary Information [file 41467_2023_38823_MOESM1_ESM.pdf]

Supplementary Information

Extreme fast charging of commercial Li-ion batteries via combined thermal switching and self-heating approaches

Yuqiang Zeng[1,4], Buyi Zhang[1,2,4], Yanbao Fu[1], Fengyu Shen[1], Qiye Zheng[1,2,3], Divya Chalise[1,2], Ruijiao Miao[1,2], Sumanjeet Kaur[1], Sean D. Lubner[1], Michael C. Tucker[1], Vincent Battaglia[1], Chris Dames[1,2], Ravi S. Prasher[1,2]*

[1]Energy Storage and Distributed Resources Division, Lawrence Berkeley National Laboratory; Berkeley, CA, 94720, USA

[2]Department of Mechanical Engineering, University of California; Berkeley, CA, 94720, USA

[3]Mechanical and Aerospace Engineering Department, The Hong Kong University of Science and Technology; Hong Kong, China

*Corresponding author: rsprasher@lbl.gov.

[4]These authors contributed equally.

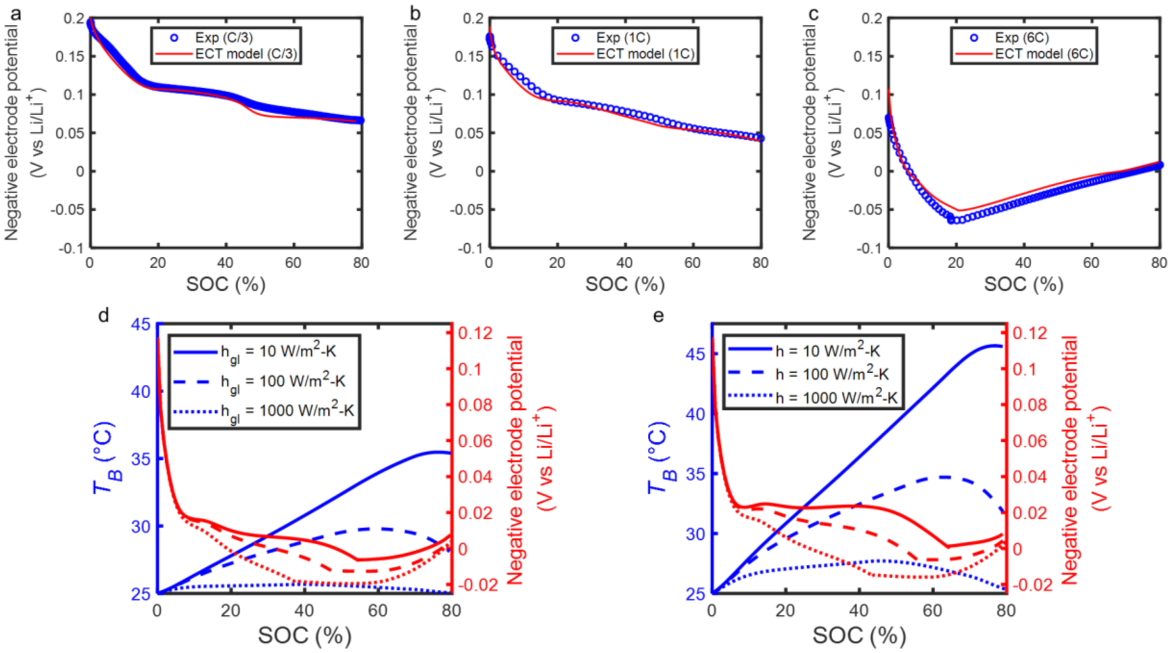

Supplementary Figure 1. Evaluation of thermal strategy using ECT model. Verification of our ECT model by comparing to the negative electrode potential measured in a three-electrode cell (*i.e.*, a 32 mAh C||LCO single-layer pouch cell using lithium foil as the reference electrode) during charging at **a**) C/3 (0.94 mA/cm^2), **b**) 1C (2.83 mA/cm^2), and **c**) 6C (16.98 mA/cm^2). Prediction of battery temperature and negative electrode potential during XFC of 10-Ah C||LCO pouch cells using **d**) the system-level thermal strategy and **e**) the local thermal control of the cell. We use the global thermal conductance per unit area (h_{gl}) ranging from 10 W/m^2-K to 1000 W/m^2-K to mimic the different status of coolant flow. Regardless of the flow status, the heat leakage from the cell to the BTMS reduces the battery temperature rise during XFC and results in the negative electrode potentials below 0 V vs Li/Li$^+$. In contrast, the minimum negative electrode potential can maintain positive by local thermal control of the cell (*i.e.*, minimized heat leakage from the battery to the BTMS) with h ~10 W/m^2-K.

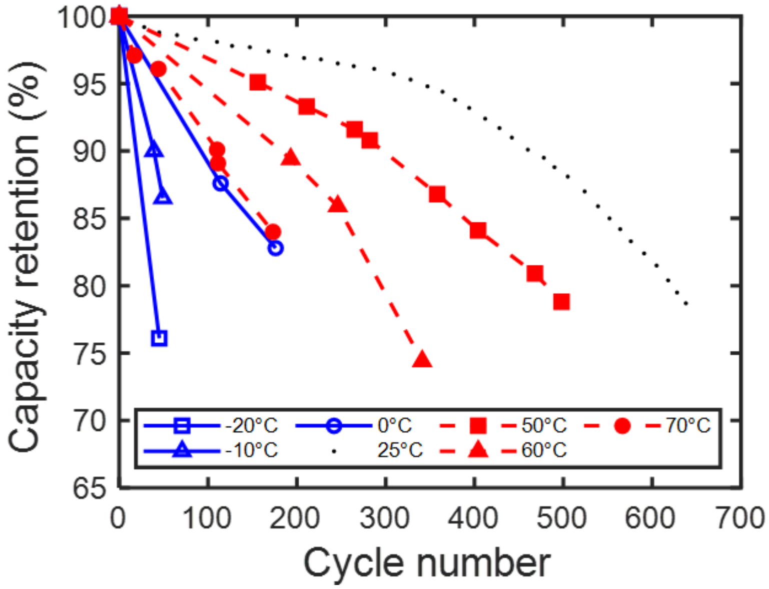

Supplementary Figure 2. Impact of operation temperature on the cycle life of LIBs[1]. Based on the experimental data collected by Waldmann *et al.*[1]**,** the optimal operation temperature is ~25 °C for cycling at slow or medium rates (*e.g.*, 1C1C cycling). Increasing the temperature leads to increased side reactions and reduced cycle life, while the cycle life decreases at low temperature due to lithium plating during charging. In the case of the insulation protocol, the average temperature is much higher than the case of thermal switch, which adversely affects the cycle life of the insulation case.

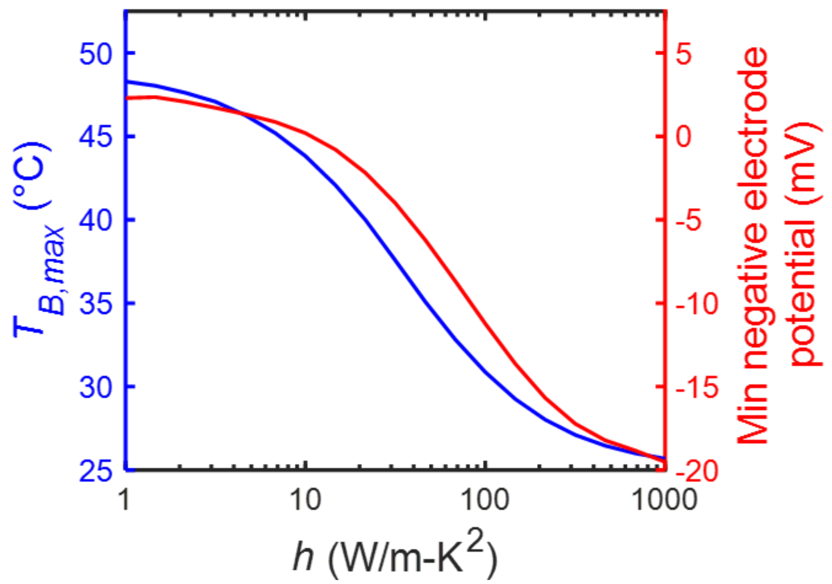

Supplementary Figure 3. Maximum battery temperature and minimum negative electrode potential during XFC using our validated ECT model as a function of thermal conductance per unit area *(h)* for the 10-Ah C||LCO pouch cell (90 mm × 61 mm × 13.5 mm). The minimum negative electrode potential was positive during XFC when the cell is thermally insulated with h ~10 W/m^2-K.

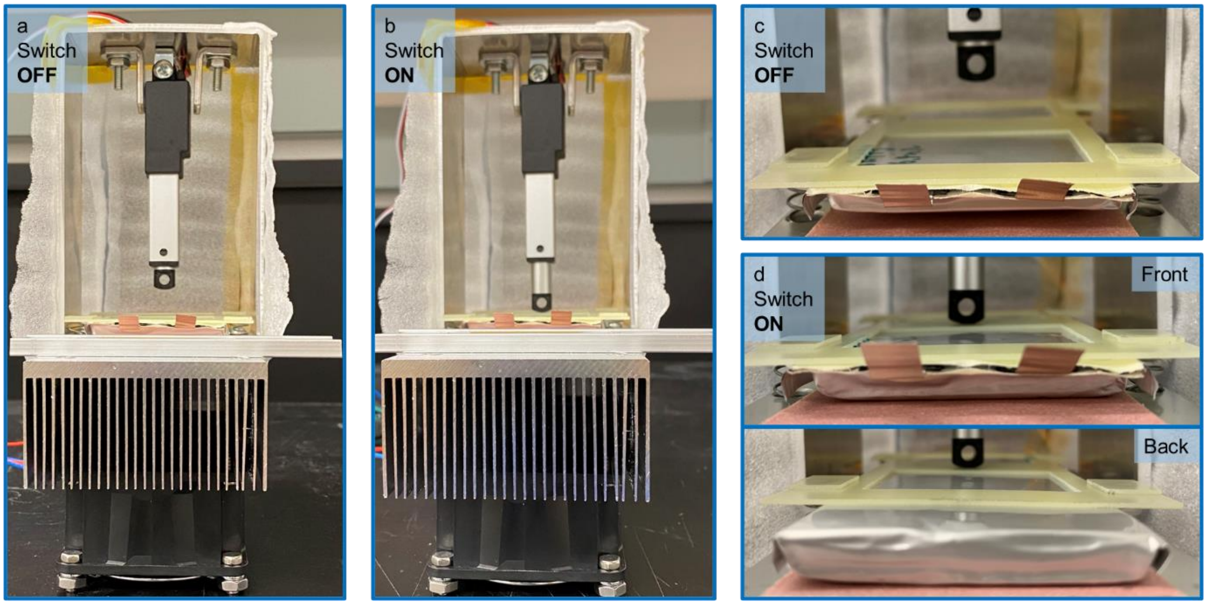

Supplementary Figure 4. Photo of the linear actuator used for ATS in the proof-of-concept study. The actuator in the original or elongated state corresponds to the switch **a**) OFF or **b**) ON, respectively. **c**, Switch OFF. A gap exists between the battery and heat sink and minimizes the heat transfer in between. **d**, Switch ON. The gap is closed as the actuator elongates and pushes the battery in contact with the heat sink.

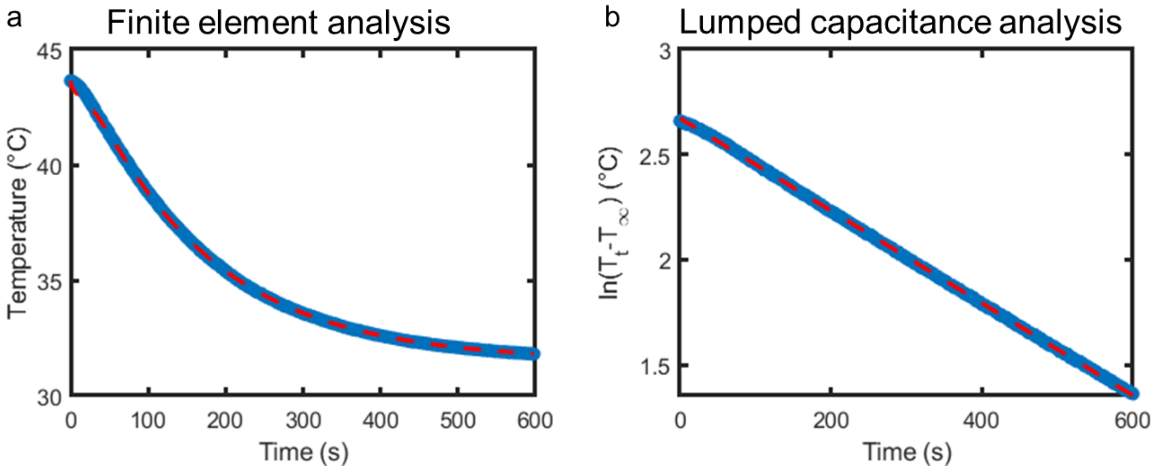

Supplementary Figure 5. Thermal analysis. a, Finite element analysis for determining the effective heat transfer coefficient (*h*) in the case of cooling and switch ON. The battery is heated in a thermal insulation state to a relatively uniform temperature and then set to a certain thermal condition (cooling or switch ON). The transient temperature during cooling is used for extracting the *h*. **b**, Lumped capacitance analysis for extracting the *h* in the case of insulation and switch OFF by fitting to the transient temperature during resting.

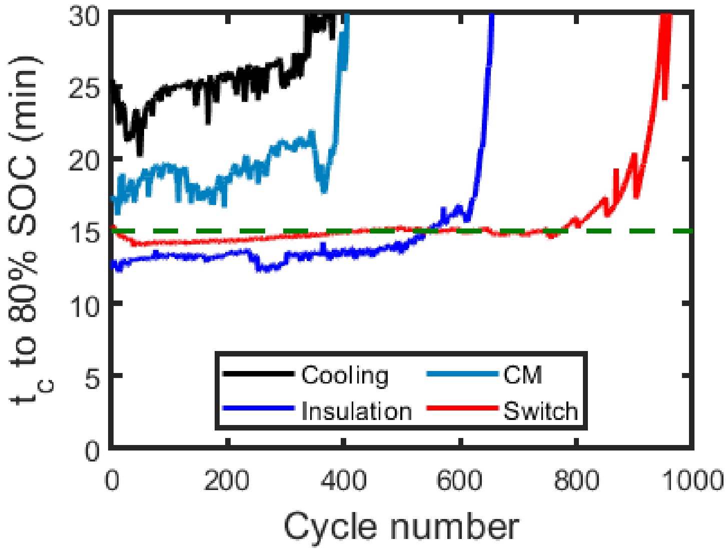

Supplementary Figure 6. Evolution of charge time with cycle number. The charge time to 80% SOC can increase to >15 min before losing 20% capacity due to battery aging.

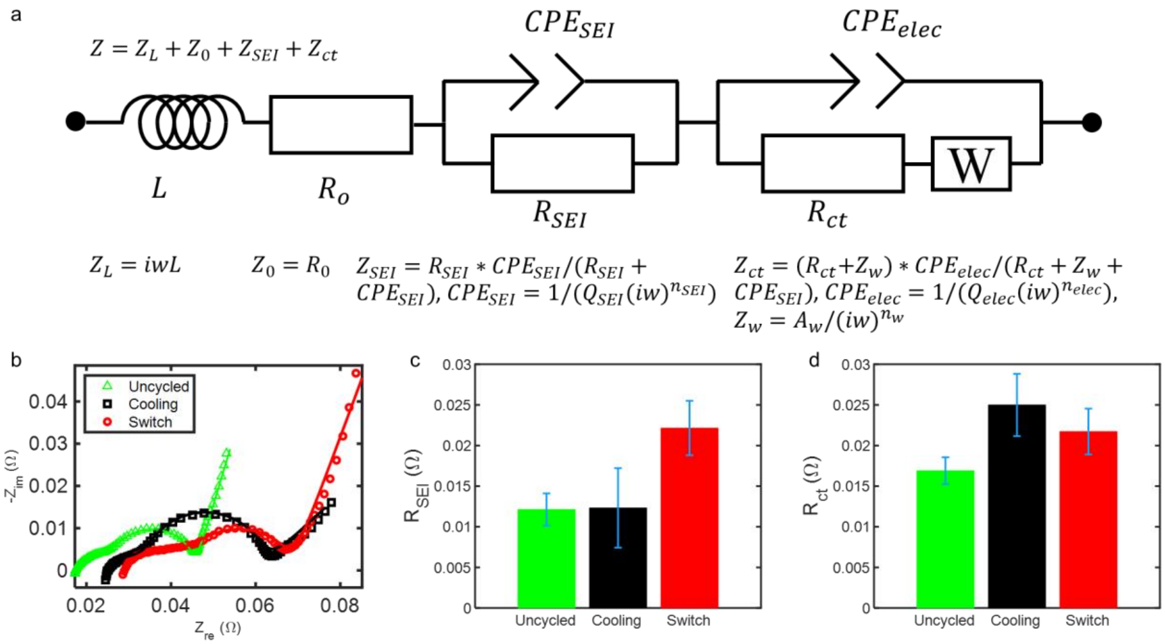

Supplementary Figure 7. EIS analysis of uncycled and aged cells. a, The equivalent circuit model[2] used for fitting the electrochemical impedance spectroscopy (EIS): R_o, R_{SEI}, and R_{ct} are the ohmic resistance, solid electrolyte interphase (SEI) layer resistance, and the charge transfer resistance, respectively. CPE_{SEI}, CPE_{elec}, and W represent the capacitance of the SEI layer, the double layer capacitance, and the Warburg diffusion element, respectively. The mathematical expressions used to fit the experimental results are given with the equivalent circuit. **b**, The EIS spectra of uncycled and aged 5-Ah C||LCO pouch cells. From the analysis, the aged cell in the case of cooling has the higher R_{ct} related to the severe lithium plating[3], and the higher R_{SEI} is observed in the case of switch associated with the longest XFC cycle life. The root of mean squared error between the experimental data (markers) and the fitting results (solid lines) are 2×10^{-4} Ω, 5.61×10^{-4} Ω, 3.95×10^{-4} Ω for Uncycled, Cooling, Switch cases, respectively. Comparison of **c**) R_{SEI} and **d**) R_{ct} in the aged cell from "cooling" and "switch" indicates the different aging mechanism: the larger R_{SEI} in the switch case corresponds to more side reaction products and the more significant increase of R_{ct} in "cooling" relates to the severe lithium plating[3]. The error bars in panels c) and d) correspond to fitting the raw EIS data with a 95% confidence interval.

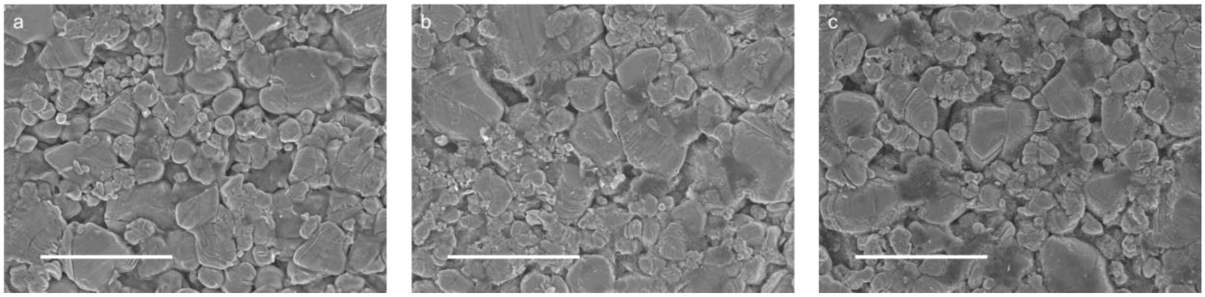

Supplementary Figure 8. SEM images of a) uncycled positive electrode and aged positive electrodes for the case of b) cooling and c) switch + CM using 5-Ah C||LCO pouch cells. No observable cracking is found in the aged **positive electrodes**, which verifies the minimal effect of **positive electrode** aging on the capacity degradation in our study[4]. The scale bar is 25 μm in these panels.

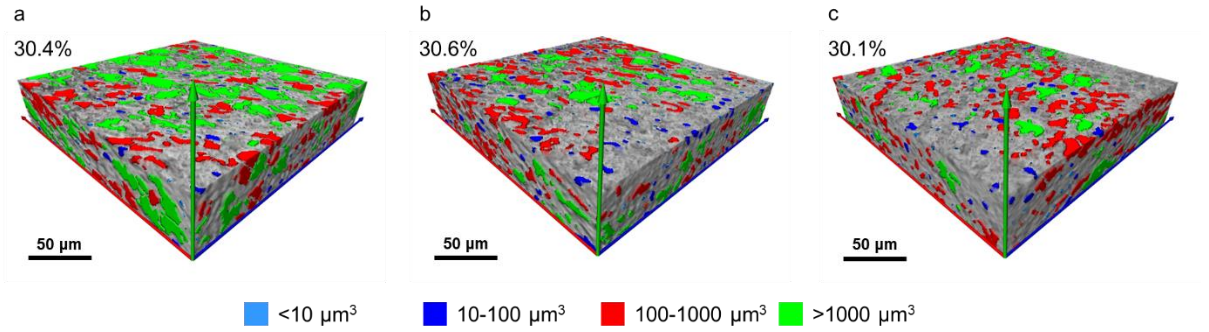

Supplementary Figure 9. Tomography of a) uncycled positive electrode and aged positive electrodes for the case of b) cooling and c) switch + CM using 5-Ah C||LCO pouch cells. The positive electrode porosity remains almost the same here, unlike the strong changes of negative electrode porosity with battery aging seen in Fig. 3 of the main text.

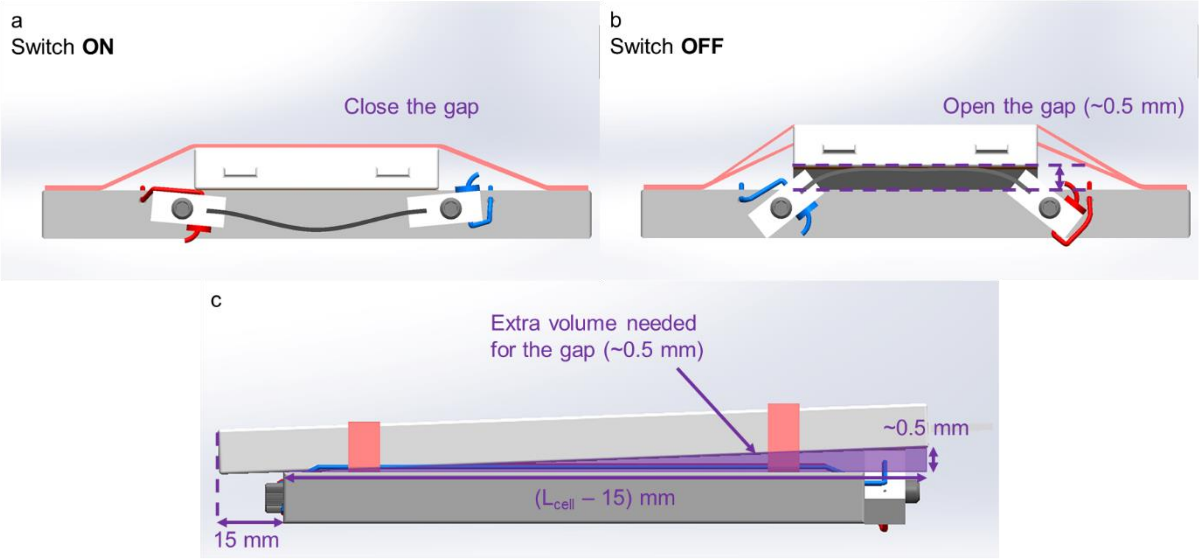

Supplementary Figure 10. Different views of the SMA-based thermal switch for a pouch cell. Front view of **a**) Switch ON and **b**) Switch OFF shows the insulating air gap used for ATS. **c**, Side view of the switch shows the volume added to the BTMS due to the presence of this air gap (shaded purple wedge shape).

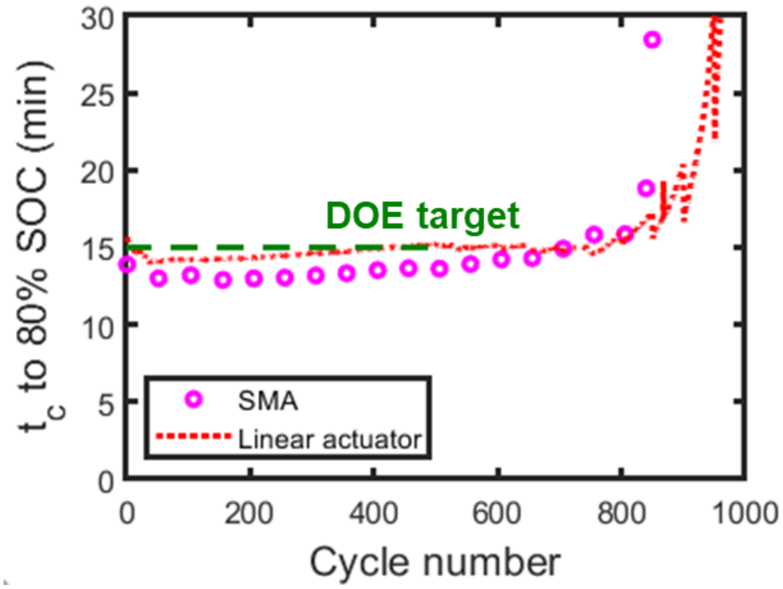

Supplementary Figure 11. Comparison of charge time with the XFC target set by US DOE (the green dashed line), *i.e.*, <15-min charge time.

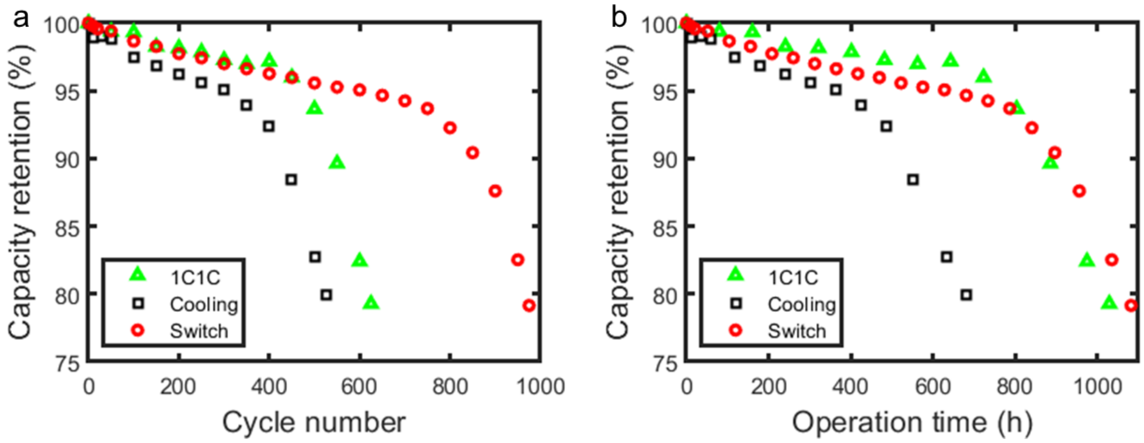

Supplementary Figure 12. Comparison of 6C1C results (black and red points) with 1C1C cycling results using the "cooling" protocol (green points). Capacity retention as a function of **a**) cycle number and **b**) operation time for 5-Ah C||LCO pouch cells. The comparable operation time of 6C1C and 1C1C cycling suggests that the degradation related to XFC is largely reduced with our TMCP. The current density corresponding to 1C and 6C rates are 2.83 mA/cm^2 and 16.98 mA/cm^2, respectively.

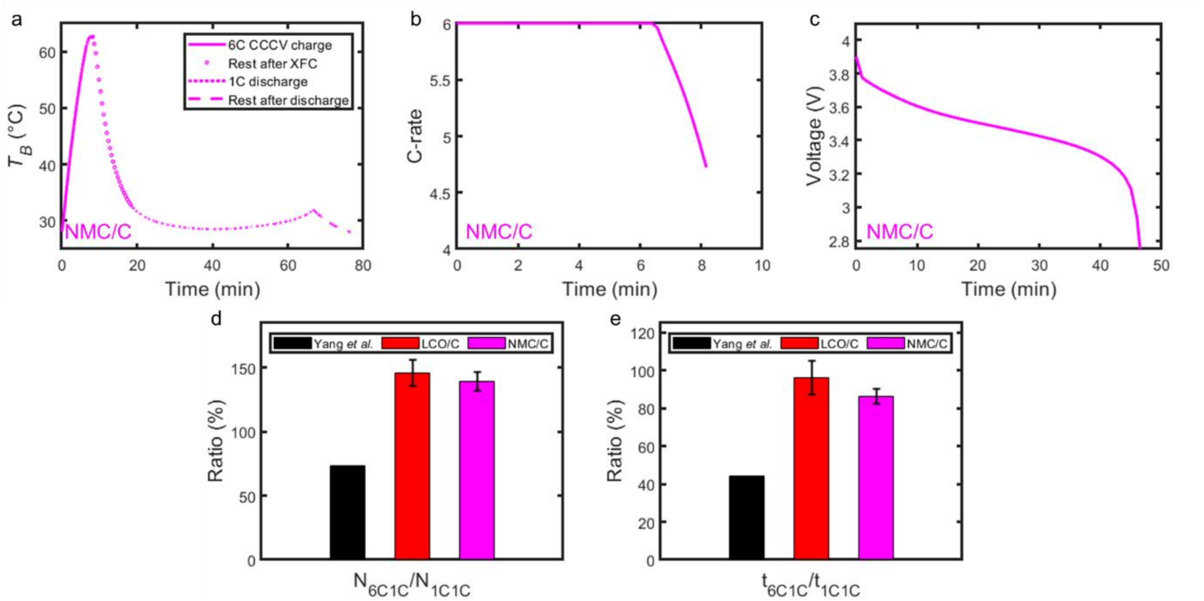

Supplementary Figure 13. XFC cycling of 10-Ah C||NMC pouch cells (model number: 10059156-5C). Representative evolution of **a**) T_B, **b**) charge rate, and **c**) discharge voltage for C||NMC cells by active thermal switching. A comparison of normalized performance, d) N_{6C1C}/N_{1C1C} and e) t_{6C1C}/t_{1C1C}, between our approach and the method by Yang *et al.*[5]. The t_{6C1C}/t_{1C1C} with our approach is ~86.2% for C||NMC cells, which is slightly lower than that for C||LCO cells (~96.2%) possibly due to the aging effect related to the higher charging temperature. Considering the high t_{6C1C}/t_{1C1C} in both types of cells compared to that in Yang *et al.*'s work[5] (44.3%), the degradation effect due to the increased charging temperature is minor even in C||NMC cells.

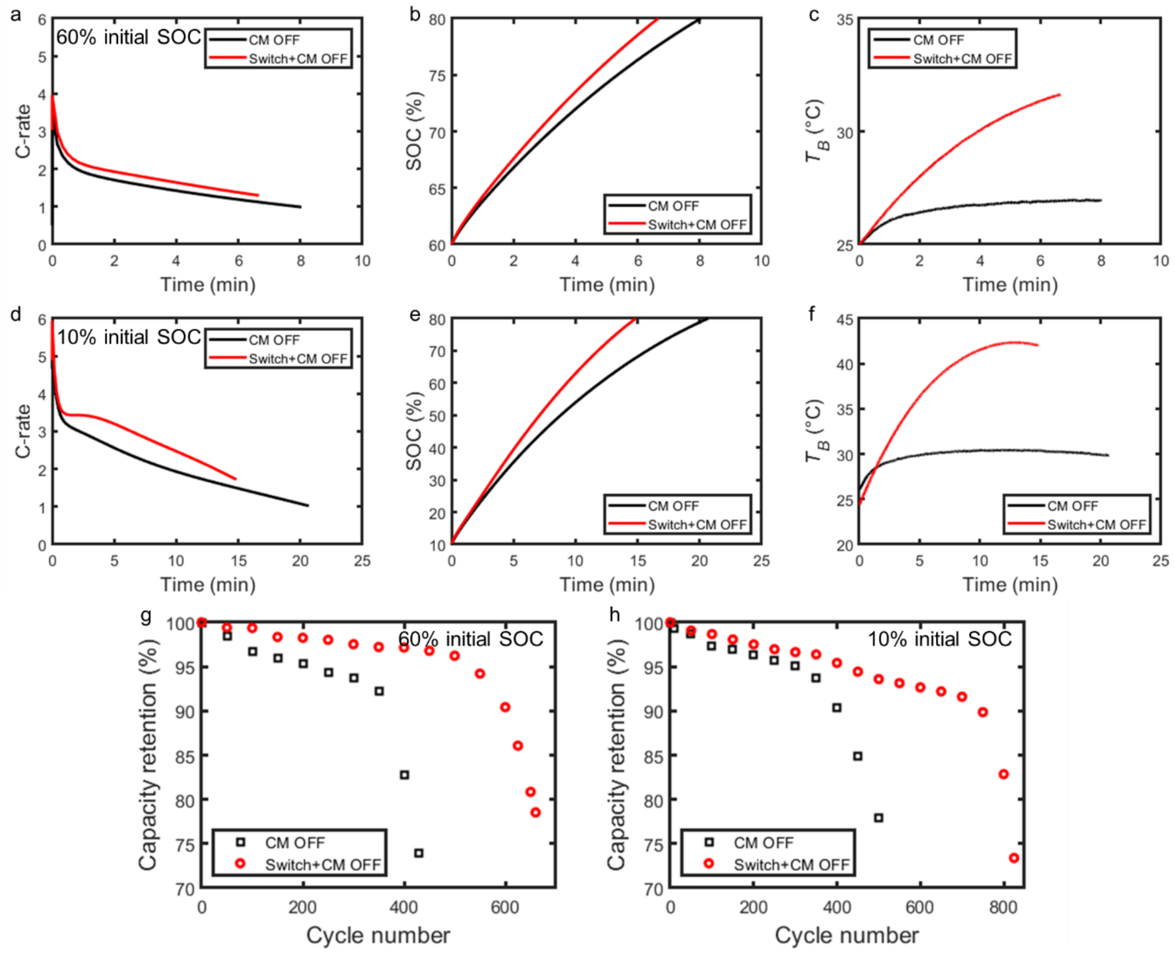

Supplementary Figure 14. XFC of 5-Ah C||LCO pouch cells from nonzero initial SOCs at T_a of 25 °C. Evolution of **a**) C-rate, **b**) SOC, and **c**) T_B from SOC = 60% to 80%. Our approach benefits the charging process even at such an unusual high initial SOC. The charge time is ~8.03 min with "CM" and reduces to ~6.66 min with our approach, and the corresponding effective C-rate is ~1.5C and ~1.8C, respectively. With a representative initial SOC like 10%, our method leads to charge time <15 min to SOC = 80%, as shown in the evolution of d) C-rate, e) SOC, and f) T_B during 6C CCCV charging. For both nonzero initial SOCs, our approach yields high CE compared to that of "CM" (99.5% vs. 98.4% for 10% initial SOC and 99.8% vs. 99.6% for 60% initial SOC). Further, the cycling performance verifies the efficacy of our strategy (see the capacity retention for the cells with **g**) 60% and **h**) 10% initial SOC).

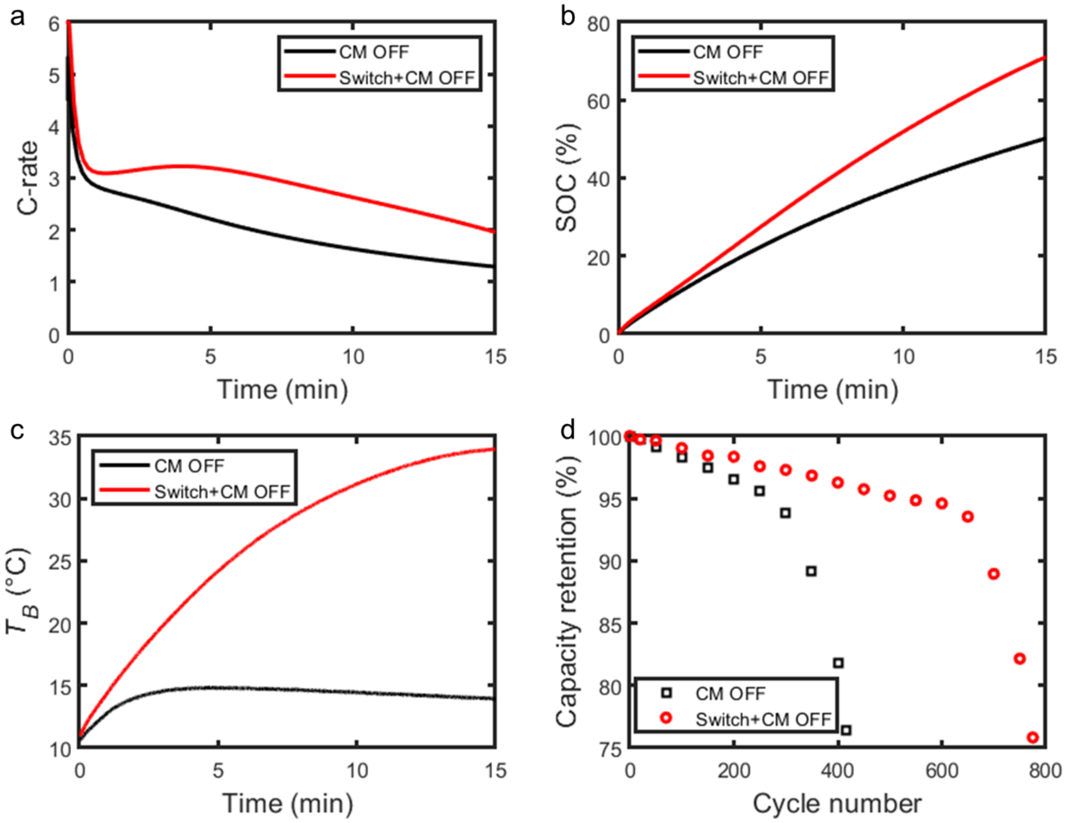

Supplementary Figure 15. XFC cycling of 5-Ah C||LCO pouch cells at T_a of 10 °C. The efficiency of retaining battery heat affects the evolution of **a**) C-rate, **b**) SOC, **c**) T_B during XFC. The SOC at 15 min in the case of CM and CM + Switch is 50.2% and 71.1%, respectively. The average CE is 95.3% for "CM" and 99.6% for "CM + Switch". The different T_B and battery kinetics leads to different XFC cycling performance, as shown in **d**) capacity retention. Note for low ambient temperature the thermal switch is in OFF state both during discharging and charging as opposed to high temperature (25 °C) where the Switch is in ON state during discharging to dissipate the heat. This retains the battery heat during discharging to increase the battery temperature before charging.

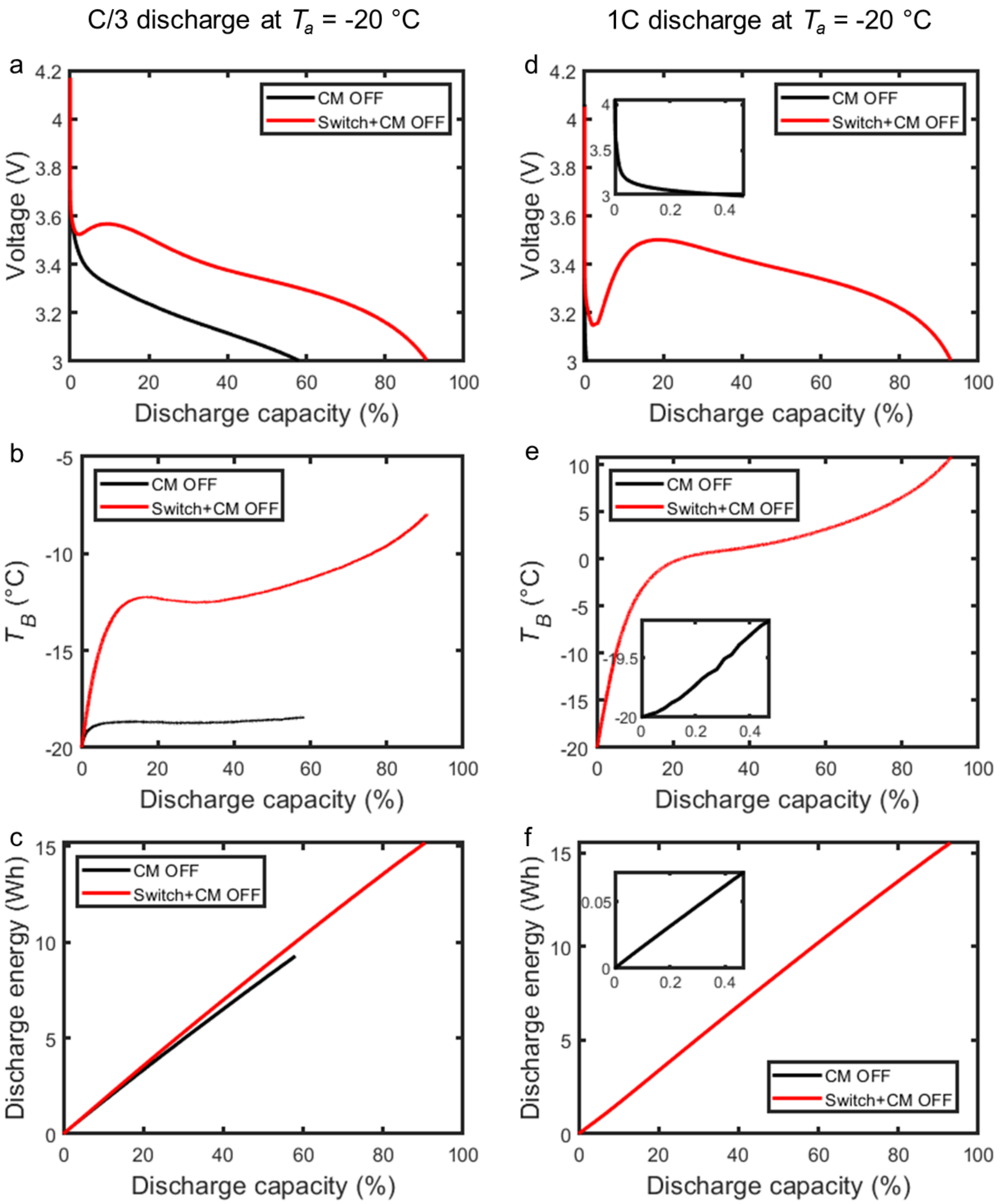

Supplementary Figure 16. Discharging of 5-Ah C||LCO pouch cells at T_a = -20 °C. At both discharge rates, the efficient retention of battery heat with our method improves the battery kinetics and leads to >70% discharge energy, as shown in the evolution of **a, d**) voltage, **b, e**) T_B, and **c, f**) discharge energy.

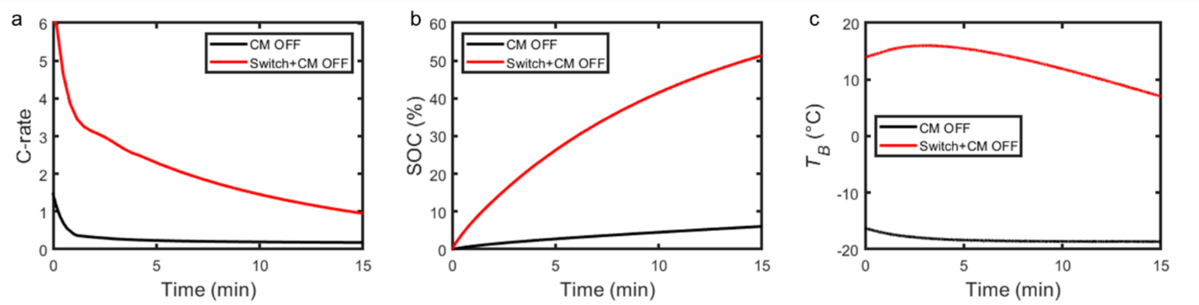

Supplementary Figure 17. XFC of 5-Ah C||LCO pouch cells after discharging at T_a = -20 °C. Evolution of **a**) C-rate, **b**) SOC, and **c**) T_B during XFC at T_a of -20 °C, after 1C discharging to 2.5 V. The different initial T_B is a result of the different thermal condition and battery temperature rise during discharging. The thermal condition also affects the rise of T_B and battery kinetics during XFC. The SOC in 15 min for "CM" and "CM + Switch" is 6.1% and 51.4%, respectively. Here, the cell is discharged at 1C to a lower cut-off voltage (2.5 V) for a reasonable low initial SOC before XFC.

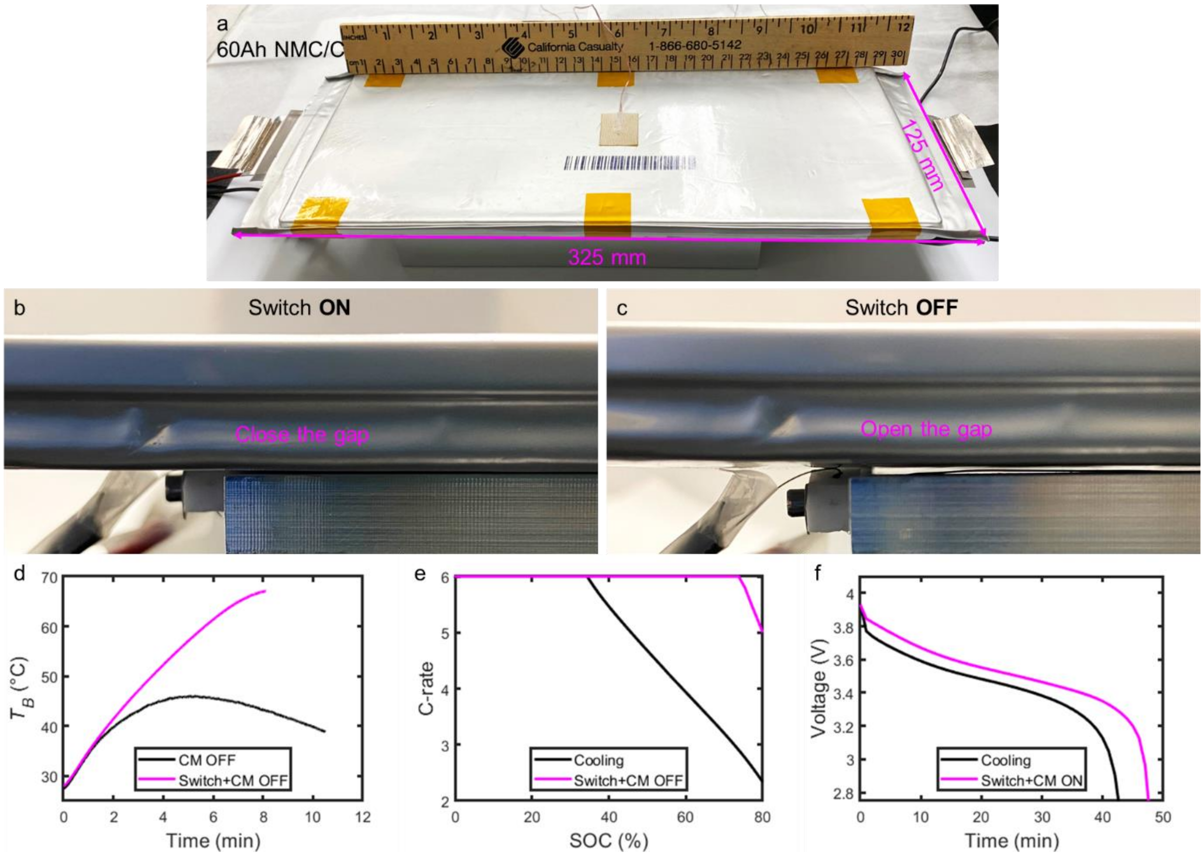

Supplementary Figure 18. Active thermal switching of large-format cells. a, SMA-based thermal switch for large-format 60-Ah C||NMC multi-layer pouch cells (L×W×H: 325 mm × 125 mm × 11.5 mm). The size of our device (*e.g.*, the SMA wire length and heat sink plate dimensions) increases accordingly with the cell geometry (see Supplementary Figure 10). Side view of **b**) Switch ON and **c**) Switch OFF shows the gap change for thermal switching. A large gap shown in **c**) is created for demonstration only, while the gap remains ~0.5 mm in all the experiments. Representative evolution of **d**) T_B, **e**) charge rate, and **f**) discharge voltage by thermal switching and coolant modulation. The high CE by thermal switching compared to that of CM (99.08% vs. 88.77%) demonstrates the efficacy of our approach for large-format cells.

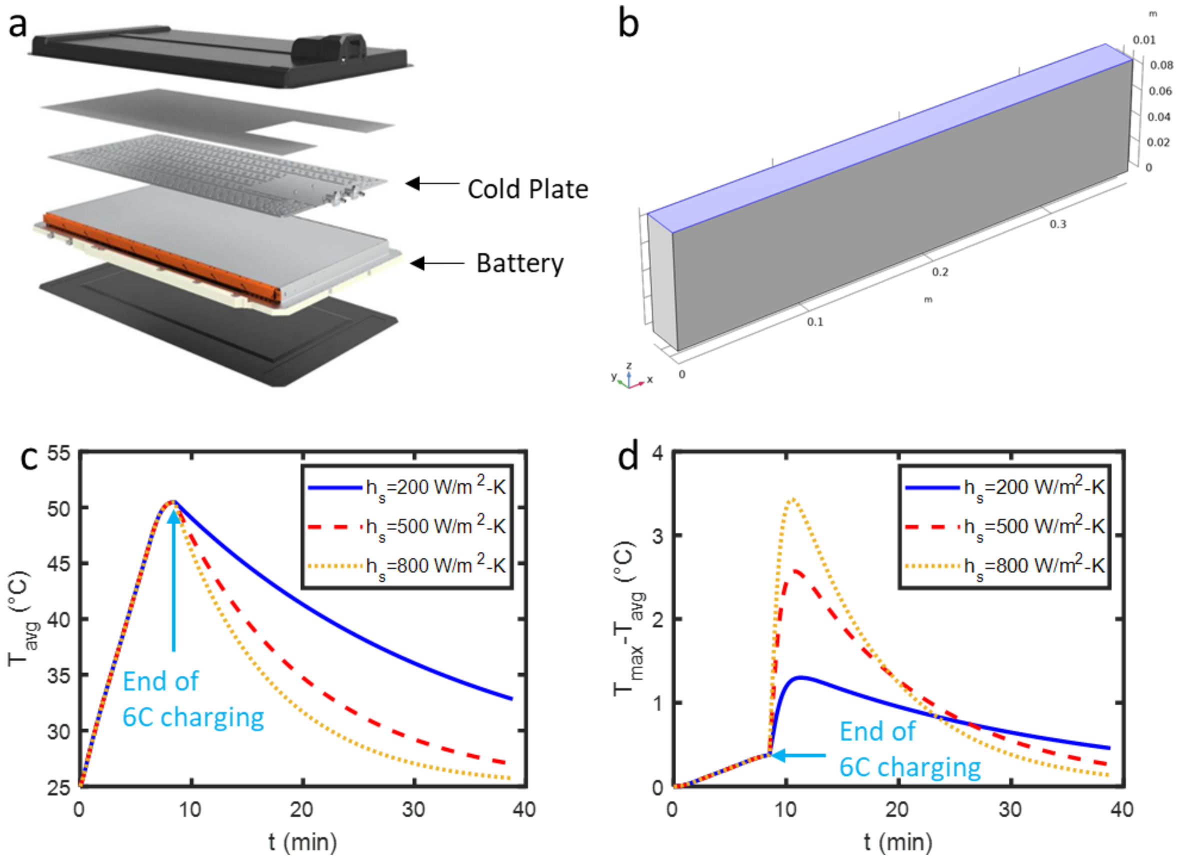

Supplementary Figure 19. Active thermal switching at the side surface. Schematics of (a) the commercial battery pack using the side surface of batteries for heating/cooling and (b) the geometry of a representative prismatic cell (36.7 cm × 8.65 cm × 2.8 cm) used in our simulation. Details on the other parameters and simulation can be found in Supplementary Note 1. For this cell, the thermal resistance associated with the front-surface heat transfer is 0.55 *K/W*, which is much higher than the thermal resistance related to the side surface (*i.e.*, 0.17 *K/W*). This is a result of the cell geometry and the high thermal conductivity anisotropy (*i.e.*, the in-plane thermal conductivity is much higher than the cross-plane thermal conductivity). Thus, the side surface is used for thermal management in this type of cell. As a verification, we performed 3D heat transfer simulation to evaluate the performance of cooling using the side surface. The heat transfer coefficient corresponding to the side surface is denoted as h_s. The predicted evolution of (c) average battery temperature and (d) temperature difference proves the effectiveness of side-surface cooling for the cell with a certain geometry. The battery was firstly 6C (~13 mA/cm^2) fast charged to 80% SOC with switch OFF which brought it to a high temperature. Then the switch was turned ON to dissipate the heat. With h_s in the range of forced convection, the battery could be cooled down to <40 °C in a short time, *e.g.*, the battery drops below 36 °C in 10 mins with $h_s = 500W/(m^2K)$.

Supplementary Tables

Supplementary Table 1. Parameters and the root of mean squared error (RMSE) between the experimental data (markers) and the fitting results (solid lines) from the EIS analysis of uncycled and aged 5Ah C||LCO pouch cells in Supplementary Figure 7b. The cells were discharged at 1C to 3.0 V and rested for ~2h before the EIS tests. The aged cells were tested after losing >20% capacity, *i.e.*, cycle 525 and 975 for the case of cooling and switch, respectively.

	Uncycled (Cycle 0)	Cooling (Cycle 525)	Switch (Cycle 975)
L (H)	8.2655×10^{-8}	7.1976×10^{-8}	8.585×10^{-8}
R_0 (Ω)	0.016787	0.023981	0.025389
R_{SEI} (Ω)	0.012135	0.012327	0.022153
R_{ct} (Ω)	0.016909	0.025	0.021727
Q_{SEI} ($s^{n_{SEI}}/\Omega$)	3.4697	2.2677	1.1057
Q_{elec} ($s^{n_{elec}}/\Omega$)	6.6994	3.0594	4.4145
A_W (Ω/s^{n_W})	1.7674×10^{-3}	3.9013×10^{-3}	3.3181×10^{-3}
n_{SEI}	0.605933	0.562289	0.498406
n_{elec}	1	0.997486	0.834558
n_W	0.817654	0.488628	0.77722
RMSE (Ω)	2×10^{-4}	5.61×10^{-4}	3.95×10^{-4}

Supplementary Table 2. Capacity loading of uncycled electrodes and aged electrodes in the switch case for 5-Ah C||LCO pouch cells.

Calibration at C/10	Uncycled Negative Electrode	Uncycled Positive Electrode	Cycled Negative Electrode		Cycled Positive Electrode	
			Edge	Center	Edge	Center
Q_c (mAh)	3.92	3.62	3.82	3.97	3.56	3.68
Q_d (mAh)	3.91	3.59	3.81	3.96	3.52	3.65
Loading (mAh/cm^2)	3.09	2.83	3.01	3.12	2.78	2.88

Supplementary Table 3. Mass, material cost, and volume (Supplementary Figure 10c) of the SMA switch and C||LCO pouch cell.

	Mass (g)	Unit cost	Material cost ($)	L×H (mm)
SMA wires	0.08	$30-300 /kg[6]	0.0024-0.024	-
Pivot blocks	0.64	$15 /kg[7]	0.0096	-
Spring steel strip	0.44	$0.25-2.70 /kg[8]	0.00011-0.0012	-
Total	1.16	-	0.012-0.035	$(L_{cell}$-15)×~0.5
5Ah C||LCO	82.36	$97 /kWh[9]	1.79	117×7.2

Supplementary Table 4. Relative mass, material cost, and volume of the SMA switch, as normalized to the 5 Ah C||LCO pouch cell.

	Relative mass (%)	Relative material cost (%)	Relative volume (%)
5Ah C||LCO	1.4	0.7-1.9	~3.0

Supplementary Table 5. Materials and properties for the three-electrode cells.

Graphite negative electrode	91.83 wt% Superior Graphite SLC 1506T<br>2 wt% Timcal C45 carbon<br>6 wt% Kureha 9300 PVDF binder<br>0.17 wt% Oxalic acid<br>Cu foil (MSE Supplies, USA; ≥99.5% purity) thickness: 10 μm<br>Coating thickness: 70 μm<br>Width × Length: 3.15 cm × 4.15 cm<br>Porosity: 38.2%<br>Coating loading: 9.38 mg/cm^2<br>Estimated areal capacity: 2.84 mAh/cm^2
LCO positive electrode	94 wt% LCO<br>2 wt% Timcal C45 carbon<br>4 wt% Solvay 5130 PVDF binder<br>Al foil (MSE Supplies, USA; ≥99.5% purity) thickness: 15 μm<br>Coating thickness: 70 μm<br>Width × Length: 3 cm × 4 cm<br>Porosity: 32.4%<br>Coating loading: 16.80 mg/cm^2<br>Estimated areal capacity: 2.67 mAh/cm^2
Liquid electrolyte	1.2 M $LiPF_6$ in EC/EMC (weight ratio 3:7), ~400 μL for each cell, from Daikin America, water content < 8 p.p.m.
Separator (double layer)	Celgard 2400 from Celgard LLC<br>Thickness per layer: 25 μm<br>Porosity: 41%

Supplementary Notes

Supplementary Note 1: Electrochemical-thermal (ECT) simulation

Electrochemical-thermal (ECT) simulations were performed in COMSOL Multiphysics 5.6. We coupled the Lithium-Ion Battery Module and the Heat Transfer Module for the simulation of battery operation in different thermal conditions, based on a Newman pseudo 2D electrochemical model and a 3D transient heat transfer model.

The following conservation equations are solved to describe the 1D electrochemical processes through the porous electrode along with the Li diffusion inside the active material particles.

Charge conservation in solid particles:

$$\nabla \cdot \left(\sigma_s^{eff} \nabla \phi_s\right) - j = 0. \tag{S1}$$

Charge conservation in electrolyte:

$$\nabla \cdot \left(\kappa^{eff} \nabla \phi_e + \kappa_D^{eff} \nabla \ln c_e\right) + j = 0, \tag{S2}$$

in which the effective diffusional ionic conductivity:

$$\kappa_D^{eff} = \frac{2RT\kappa^{eff}}{F}(t^+ - 1)(1 + \frac{d \ln f_\pm}{d \ln c_e}). \tag{S3}$$

Material conservation in electrolyte:

$$\varepsilon \frac{\partial c_e}{\partial t} = \nabla \cdot \left(D_e^{eff} \nabla c_e\right) + \frac{1-t^+}{F} j. \tag{S4}$$

Material conservation in solid particles is governed by the Fick's law in sphere:

$$\frac{\partial c_s}{\partial t} = \frac{1}{r^2} \frac{\partial}{\partial r}(D_s r^2 \frac{\partial c_s}{\partial r}), \tag{S5}$$

with boundary condition on particle surface:

$$-D_{s,i} \frac{\partial c_{s,i}}{\partial r}|_{r=R_i} = \frac{i}{F}. \tag{S6}$$

Butler-Volmer equation for charge transfer kinetics relating the reaction current (i) with the surface overpotential:

$$i = i_0[\exp\left(\frac{\alpha_a F}{RT}\eta\right) - \exp\left(-\frac{\alpha_c F}{RT}\eta\right)], \tag{S7}$$

in which the kinetic overpotential:

$$\eta = \phi_s - \phi_e - U_i\left(c_{s,i}\right) - iR_f, \tag{S8}$$

and exchange current density:

$$i_0 = k(T) c_{s,i}^{\alpha_c} c_e^{\alpha_a} (c_{s,max} - c_{s,i})^{\alpha_a}. \tag{S9}$$

The reaction current density on particle surface and volumetric current density in the electrodes are related:

$$j = ai, a = 3 * (1 - \varepsilon)/r_i. \tag{S10}$$

The heat generation power:

$$q = j(\phi_s - \phi_e - U) + \sigma_s^{eff} \nabla\phi_s \cdot \nabla\phi_s + \kappa^{eff} \nabla\phi_e \cdot \nabla\phi_e + \kappa_D^{eff} \nabla \ln c_e \cdot \nabla\phi_e + j(T\frac{dU}{dT}). \tag{S11}$$

The heat generation comes from four terms: $j(\phi_s - \phi_e - U)$ represents kinetic heat, $\sigma_s^{eff} \nabla\phi_s \cdot \nabla\phi_s, \kappa^{eff} \nabla\phi_e \cdot \nabla\phi_e$ and $\kappa_D^{eff} \nabla \ln c_e \cdot \nabla\phi_e$ are joule heat from electronic resistance, ionic resistance and concentration overpotential respectively, and $j(T\frac{dU}{dT})$ is the reversible heat.

To verify our ECT model, we simulated the negative electrode potential during XFC and observed a good agreement with the experiment results from a three-electrode cell (see

Supplementary Figures 1a-c). We assembled three-electrode cells (*i.e.*, 32 mAh C||LCO single-layer pouch cells using lithium foil as the reference electrode) for the verification study[10].

For the ECT simulation of a representative commercial battery, *e.g.*, commercial C||LCO cells, the thermal model uses the heat generation rate from the electrochemical simulation as input, and the evolution of battery temperature during charging in different thermal conditions is calculated. We performed 3D transient heat transfer simulations for the battery with or without BTMS. The temperature distribution inside the battery (T_B) is governed by the Fourier's law:

$$\rho C_p \frac{\partial T_B}{\partial t} = \frac{\partial}{\partial x}\left(k_x \frac{\partial T_B}{\partial x}\right) + \frac{\partial}{\partial y}\left(k_y \frac{\partial T_B}{\partial y}\right) + \frac{\partial}{\partial z}\left(k_z \frac{\partial T_B}{\partial z}\right) + q, \quad \text{(S12)}$$

where ρ, C_p, k, and q are the density, heat capacity, thermal conductivity, and volumetric heat generation rate of the battery, respectively. This equation was solved with the coupled heat generation rate and the convective heat transfer boundary condition on the surface. Details on the calculation of heat generation rate are shown in Equation S11[11,12]. For the simulation of cell packs including BTMS, the BTMS was an additional thermal mass resulting in heat transfer with the battery (*e.g.*, heat leakage from the battery to the heat sink). According to Yang *et al.*[11], the gravimetric cell-to-pack ratio is 55-65%, which means 35-45% of the pack weight is taken by management system, metals, cabling and others. We estimate the heat capacity of management system as the average of aluminum and coolants (water/glycol)[13].

Gen2 electrolyte (1.2 M $LiPF_6$ in EC:EMC 3:7) was used in all the studies, which is the baseline electrolyte for XFC suggested by the US DOE. We used the transport properties of electrolyte, graphite and $LiCoO_2$, *e.g.*, diffusion coefficient and conductivity, provided by the COMSOL material library. The open circuit voltage (OCV) of graphite and $LiCoO_2$ were also from the COMSOL material library.

A representative 3D temperature distribution at the end of 6C (~13 mA/cm^2) charge when h = 10 W/m^2K

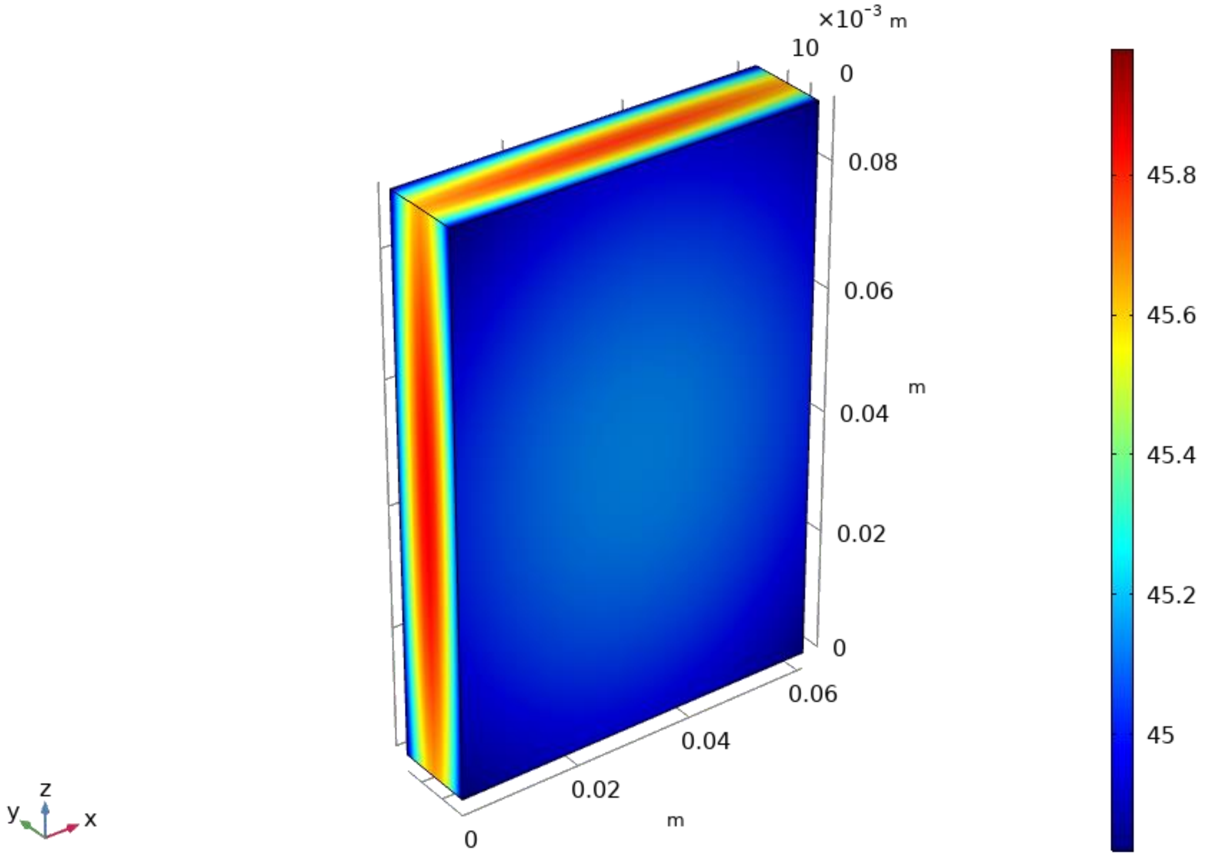

Electrode

Parameter	Negative electrode (Graphite)	Positive electrode ($LiCoO_2$)
Thickness (μm)	55.4	52.2
Initial Porosity	0.382	0.356
Loading (mAh/cm^2)	2.34	2.11
Particle radius (μm)	5	5[14]
Specific surface area (m^{-1})	3.267×10^5	2.97×10^5
Usable capacity range by lithium intercalation fraction	0.12~0.95	0.453~0.994
Bruggeman exponent, p	2.55	2.2

Reference exchange current density (A/m^2)	2.1[14]	2.1
Activation energy of exchange current density (kJ/mol)	68[14]	69[15]
Solid-state diffusivity, $D_s(cm^2/s)$	COMSOL Materials Library	COMSOL Materials Library
Activation energy of solid-state diffusivity (kJ/mol)		25[16]

Separator

Electrolyte concentration (mol/L)	1.2
Thickness (μm)	25
Porosity	0.41
Bruggeman exponent, p	2

Cell

Positive Electrode Material	$LiCoO_2$
Specific heat of cell, c_p ($J/(kg * K)$)	1100[17]
Mass of the battery (kg)	0.211
Thickness (mm)	13.5
Length (mm)	90
Width (mm)	61
In-plane thermal conductivity (W/(m*K))	25[12]
Cross-plane thermal conductivity (W/(m*K))	0.8[12]

Supplementary Note 2: Thermal Analysis

Effective thermal conductance per unit area (h) was obtained from the experiments by matching the temperature profile (Fig. 1d) from the experiment and detailed thermal model in COMSOL (Supplementary Figure 5).

Supplementary Note 3: Estimation of mass, volume, and material cost for the SMA switch

Each SMA device, as shown in Fig. 4a, consists of 2 SMA wires (Nitinol alloy), 1 spring steel strip (1095 spring steel), and 2 pivot blocks (Teflon). Supplementary Table 3 summarizes the mass, volume, and material cost of each component. For the estimation of volume, the volume of the strip and pivot blocks is not regarded as extra as they are built on the sidewall of the heat sink plate (*i.e.*, this space is already considered unusable in an existing BTMS). For this reason, only the volume related to the gap that affects the cell-to-pack volumetric ratio is considered in the analysis (Supplementary Figure 10c). We estimate the relative mass, volume, and material cost of the SMA switch compared to that of batteries (Supplementary Table 4). Compared to 5Ah C||LCO pouch cells, the relative mass, volume, and material cost of the SMA device is 1.4%, ~3.0%, and 0.7-1.9%, respectively. These ratios could reduce as a battery of higher capacity is used due to the increased battery mass, volume, and cost.

Supplementary Note 4: Equivalent Circuit for EIS

Supplementary Figure 7a shows the equivalent circuit model[2] used for fitting the electrochemical impedance spectra: R_o, R_{SEI}, and R_{ct} are the ohmic resistance, solid electrolyte interphase (SEI) layer resistance, and the charge transfer resistance, respectively. CPE_{SEI}, CPE_{elec}, and W represent the capacitance of the SEI layer, the double layer capacitance, and the Warburg diffusion element, respectively. The first component is an inductance, which comes from the stacks of current collectors and connecting wires.

$$Z_L = iwL. \quad \text{(S13)}$$

The second term is the ohmic resistance of the battery, mainly comprised of the electrolyte, electrode and contact resistances.

$$Z_0 = R_0. \quad \text{(S14)}$$

The third component is a resistor in parallel with a constant phase element; this one represents the impedance from the solid-electrolyte interface.

$$Z_{SEI} = R_{SEI} * CPE_{SEI}/(R_{SEI} + CPE_{SEI}), \quad \text{(S15)}$$

where $CPE_{SEI} = 1/(Q_{SEI}(iw)^{n_{SEI}})$.

The fourth component stands for the charge transfer process at the active material surfaces.

$$Z_{ct} = (R_{ct} + Z_w) * CPE_{elec}/(R_{ct} + Z_w + CPE_{SEI}), \quad \text{(S16)}$$

where $CPE_{elec} = 1/(Q_{elec}(iw)^{n_{elec}})$ and the lithium diffusion impedance $Z_w = A_w/(iw)^{n_w}$.

The total impedance equals to the sum of the four components. The parameters summarized in Supplementary Table 1 were determined by fitting this model to the EIS data (Supplementary Figure 7b).

Supplementary References

1. Waldmann, T., Wilka, M., Kasper, M., Fleischhammer, M. & Wohlfahrt-Mehrens, M. Temperature dependent ageing mechanisms in Lithium-ion batteries - A Post-Mortem study. *J. Power Sources* **262**, 129–135 (2014).

2. Choi, W., Shin, H.-C., Kim, J. M., Choi, J.-Y. & Yoon, W.-S. Modeling and Applications of Electrochemical Impedance Spectroscopy (EIS) for Lithium-ion Batteries. *J. Electrochem. Sci. Technol.* **11**, 1–13 (2020).

3. Harting, N., Wolff, N. & Krewer, U. Identification of Lithium Plating in Lithium-Ion Batteries using Nonlinear Frequency Response Analysis (NFRA). *Electrochim. Acta* **281**, 378–385 (2018).

4. Tanim, T. R. *et al.* Extended cycle life implications of fast charging for lithium-ion battery cathode. *Energy Storage Mater.* **41**, 656–666 (2021).

5. Yang, X. G. *et al.* Asymmetric Temperature Modulation for Extreme Fast Charging of Lithium-Ion Batteries. *Joule* **3**, 3002–3019 (2019).

6. Hao, M., Li, J., Park, S., Moura, S. & Dames, C. Efficient thermal management of Li-ion batteries with a passive interfacial thermal regulator based on a shape memory alloy. *Nat. Energy* **3**, 899–906 (2018).

7. Polytetrafluoroethylene (PTFE) Market by Form, End-Use Industry and Region | COVID-19 Impact Analysis | MarketsandMarkets. https://www.marketsandmarkets.com/Market-Reports/polytetrafluoroethylene-market-22472807.html.

8. Table 9. Producer price indexes for commodity and service groupings and individual items, not seasonally adjusted. https://www.bls.gov/web/ppi/ppitable09.pdf.

9. Battery Pack Prices Fall to an Average of $132/kWh, But Rising Commodity Prices Start to Bite | BloombergNEF. https://about.bnef.com/blog/battery-pack-prices-fall-to-an-average-of-132-kwh-but-rising-commodity-prices-start-to-bite/#_ftnref1.

10. Zeng, Y. *et al.* Operando spatial mapping of lithium concentration using thermal-wave sensing. *Joule* **5**, 2195–2210 (2021).

11. Yang, X. G., Liu, T. & Wang, C. Y. Thermally modulated lithium iron phosphate batteries for mass-market electric vehicles. *Nat. Energy* **6**, 176–185 (2021).

12. Zeng, Y., Chalise, D., Lubner, S. D., Kaur, S. & Prasher, R. S. A review of thermal physics and management inside lithium-ion batteries for high energy density and fast charging. *Energy Storage Mater.* **41**, 264–288 (2021).

13. Kim, G.-H. & Pesaran, A. Battery Thermal Management and Design. *World Electr. Veh. Assoc. J.* **1**, 1–61 (2007).

14. Yang, X. G. & Wang, C. Y. Understanding the trilemma of fast charging, energy density and cycle life of lithium-ion batteries. *J. Power Sources* **402**, 489–498 (2018).

15. Qiu, X. Y. *et al.* Electrochemical and electronic properties of LiCoO 2 cathode investigated by galvanostatic cycling and EIS. *Phys. Chem. Chem. Phys.* **14**, 2617–2630 (2012).

16. Okubo, M., Tanaka, Y., Zhou, H., Kudo, T. & Honma, I. Determination of activation energy for Li ion diffusion in electrodes. *J. Phys. Chem. B* **113**, 2840–2847 (2009).

17. Feng, X., Weng, C., Ouyang, M. & Sun, J. Online internal short circuit detection for a large format

lithium ion battery. *Appl. Energy* **161**, 168–180 (2016).
